# The Lyme Disease Debate Host Biodiversity and Human Disease Risk

**DOI:** 10.1289/ehp.121-a120

**Published:** 2013-04-01

**Authors:** Sharon Levy

**Affiliations:** **Sharon Levy**, based in Humboldt County, CA, has covered ecology, evolution, and environmental science since 1993. She is the author of *Once and Future Giants: What Ice Age Extinctions Tell Us about the Fate of Earth’s Largest Animals*.

In the mid-1970s, several children in the town of Lyme, Connecticut, began to suffer from painful, swollen joints. They were diagnosed with juvenile rheumatoid arthritis, a rare condition. Clinicians at Yale School of Medicine suspected the cluster of cases was caused by an infectious agent.^1^ The illness, dubbed Lyme disease, was soon recorded in an increasing number of patients in the northeastern United States. Symptoms included rashes, fevers, joint and muscle pain, and heart and neurological problems.

National Institutes of Health researcher Willy Burgdorfer identified the culprit in 1982: a spirochete bacterium that, in electron micrograph images, resembles a broken twist of barbed wire.^2^ The spirochete, named *Borrelia burgdorferi* (Bb), was first isolated from the gut of ticks collected in woodlands on Shelter Island, New York, where Lyme disease had become endemic.

**Figure:**
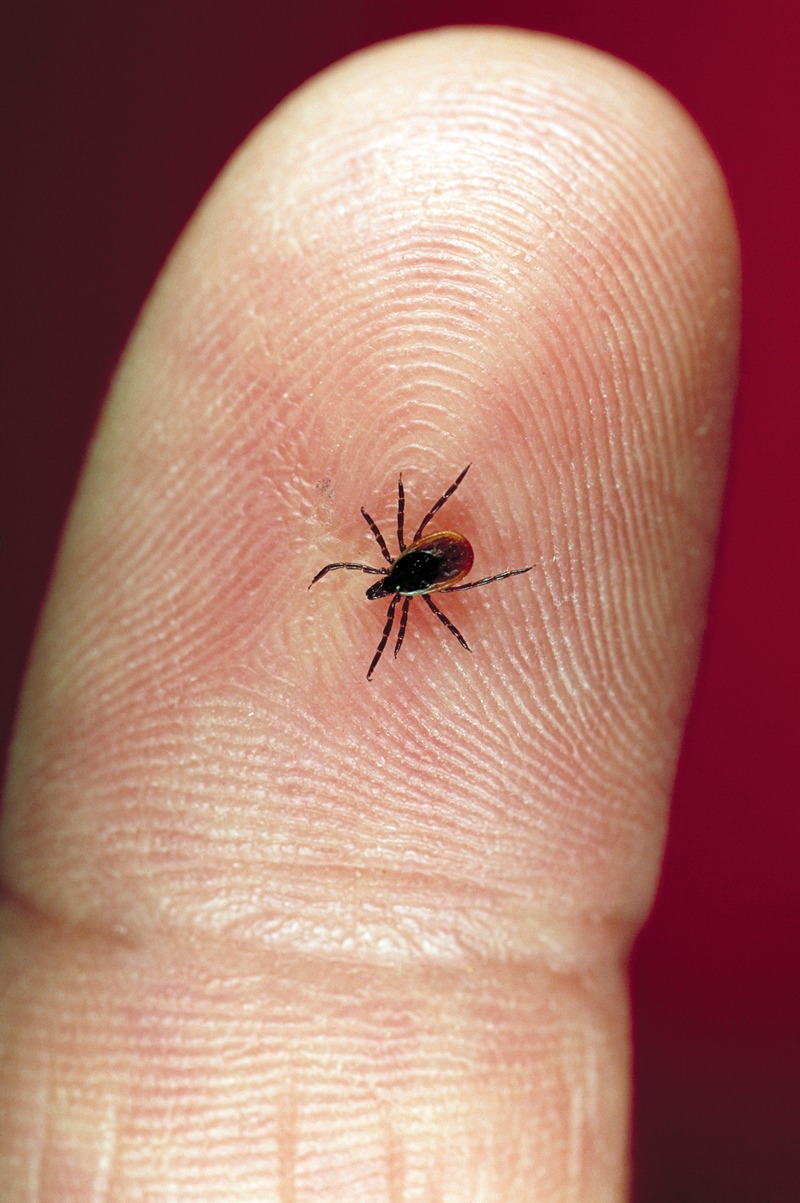
The deer tick (*Ixodes scapularis*) transmits the bacterium Borrelia burgdorfi (Bb) to humans in the course of one of three blood meals it will take in its lifetime. © Scott Camazine/Science Source

Thirty years after its discovery, Lyme disease has become by far the most common vectorborne infection in North America.^3^ Today the ecology of Bb is the subject of both intense study and intense scientific debate. Some researchers think that protecting large tracts of forest habitat—a strategy that increases the diversity of vertebrate hosts for both Bb and its tick vectors—will ultimately reduce the risk of human infection. Others argue that there is no predictable relationship between host biodiversity and human disease risk.

## History of Lyme Disease

Lyme disease occurs in Europe and Asia as well as North America, always spread by ticks in the genus *Ixodes*.^4^ Over the last decade, about 20,000–30,000 U.S. cases of Lyme disease have been reported annually by the Centers for Disease Control and Prevention, the majority occurring in the Northeast and the Midwest, where the vector is the black-legged tick, *Ixodes scapularis*.^5^ Average annual numbers of cases in Europe and Asia have been estimated at 65,467 and 3,450, respectively.^4^

The infection’s sudden rise in the United States in the 1970s gave the impression that Lyme disease was caused by a newly invading pathogen, but the diaries of early American settlers reported abundant ticks, and the evidence now shows that Bb is an ancient infection in North America. Distinctive Bb genes have been identified in museum collections of ticks from the 1940s^6^ and of white-footed mice from the turn of the twentieth century,^7^ and studies of genetic variation in separate populations of Bb suggest the pathogen existed across much of the present-day United States many thousands of years before European settlement.^8^ Nevertheless, genetic analyses indicate that this genus of bacteria originated in Europe.^9^

Bb is a microbe of forest habitats, and its history is tied to human land use. As European settlers moved west across the United States, they cleared great swaths of forest. Deer, one of the major hosts for black-legged ticks, were overhunted and dwindled to a few small, scattered populations. Populations of white-footed mice, an important reservoir host for Bb, also declined. But in some undisturbed spots in the Northeast and the Midwest, deer, white-footed mice, their tick parasites, and Bb all survived. With the abandonment of most northeastern farm fields in the mid-nineteenth century, forests regenerated, and the microbe traveled with its tick and vertebrate hosts into newly re-expanding habitats.^10^

Lyme disease now appears to be expanding outward from long-time refuges.^10^ Migratory birds carry ticks to new habitats, enabling the spread of both ticks and Bb south and north.^11^ Some bird species that host ticks are expanding their ranges north, and studies of emerging Lyme disease in Quebec, Canada, suggest that climate change makes it possible for tick vectors to survive in an area that once would have been too cold.^12^

Bb is hitched to the life cycle of its tick vector. Over the course of a life span that lasts at least two years, *Ixodes* ticks must take a blood meal from a vertebrate host on three separate occasions, dropping off the host after each meal. Tiny larval ticks hatch out on the forest floor in summer and latch onto passing hosts; because the larva waits for a host (“quests”) close to the ground, it can attach to an animal of any size, from a rodent, to a bird, to a deer. The blood from this first host will fuel the larva’s metamorphosis to the next, nymphal life stage. Nymphal ticks, no larger than a poppy seed, must take another blood meal before molting into adult form. Adult ticks drink blood from a third and final host in order to reproduce. Nymphs and adults sit higher on the vegetation to quest, so they can attach only to larger animals; this is why deer are so important for maintaining tick populations, according to Sarah Randolph, a parasite ecologist at Oxford University.

Adult ticks are large enough to be noticed by any humans they bite within the 24 hours or so it takes to pass along an infection.^13^ But nymphs are not as easily detected, and Lyme disease most often arises when a person is bitten by an infected nymph.^14^^,^^15^ Since Bb is not passed from mother ticks to their offspring, every larva comes into the world uninfected. The natural transmission cycle begins anew when a larval tick feeds on blood from an infected host, typically a mouse, chipmunk, or shrew. Once the larva develops successfully into a nymph, it will seek a new host, putting any passing humans at risk.

## Lyme Hosts

Blame for the emergence of both black-legged ticks and Lyme disease has typically focused on deer, which have abundantly repopulated the northeastern and midwestern United States over the last few decades. Yet deer turn out to be immune to infection with Bb;^16^ even though they’re an important host for ticks, especially in the adult life phase, they don’t transmit Lyme disease.

Early research tested the assumption that reducing deer populations would lower the risk of human infection by reducing numbers of infected nymphal ticks searching for a host. The results were mixed. Some studies showed a strong relationship between deer abundance and tick density.^17^^,^^18^^,^^19^ Others, however, reported that tick density was tightly linked with numbers of white-footed mice^20^ or small mammalian predators,^21^ not deer. Experiments in the Italian Alps reported an increased density of questing nymphs in habitat patches where deer had been fenced out.^22^

In assessing such findings, it is essential to take into account the time scale, says Randolph. “We all know that tick abundance will increase at first in the absence of hosts; they accumulate on the vegetation with no hosts to attach to,” she explains. “But later the abundance declines fast as the ticks die and are not replaced through natural reproduction—no hosts to feed adult ticks, no eggs.”

A number of studies in Europe and the United States have shown that while some species are competent reservoir hosts for Bb (that is, they’re likely to pass Bb along to the ticks that bite them), others are not.^23^^,^^24^^,^^25^^,^^26^^,^^27^^,^^28^^,^^29^ In 1990 Durland Fish, an epidemiologist at Yale School of Public Health, coauthored a study in which wild raccoons, striped skunks, opossums, and white-footed mice were held in cages over water pans that collected all the engorged larval ticks that dropped off. In the laboratory, the larval ticks were incubated, and the researchers tracked the numbers that developed successfully into nymphs. They then tallied the percentage of nymphs that carried Bb. Forty percent of the nymphal ticks that had fed on white-footed mice as larvae were infected. The figures for ticks that had fed on raccoons and skunks were much lower. (In the jargon of zoonoses, such animals may be “dilution hosts,” meaning they tend to make infection less prevalent in the tick population.) None of the nymphs from larvae that had fed on opossums survived long enough to be tested.^29^

In later work Richard Ostfeld, a disease ecologist at the Cary Institute of Ecosystem Studies in Millbrook, New York, and his colleagues conducted a similar experiment with larval ticks collected from white-footed mice, chipmunks, deer, and four species of songbirds. They found white-footed mice to be much more competent reservoir hosts than the other species tested.^30^ In separate work they also reported shrews to be highly competent reservoir hosts for Bb.^31^

**Figure:**
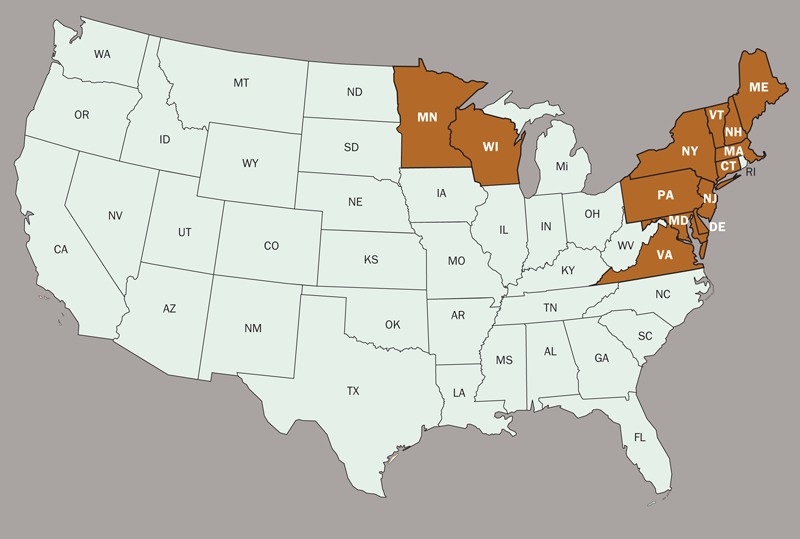
In 2011, the latest year for which statistics are available, 96% of Lyme disease cases were reported in 13 states: Connecticut, Delaware, Maine, Maryland, Massachusetts, Minnesota, New Hampshire, New Jersey, New York, Pennsylvania, Verrmont, Virginia, and Wisconsin.^5^

In a study led by Felicia Keesing, a biologist at Bard College, the team captured six species of forest animals, held them in the laboratory long enough for any ticks they’d picked up in the wild to detach, and then reinfested them with tick larvae. They found that while almost half the larval ticks placed on white-footed mice fed to repletion, only 3.5% of those on opossums fed successfully.^32^ Veeries, catbirds, chipmunks, and squirrels fell between these two extremes as tick hosts. The authors noted that confinement of the animals to the laboratory could have affected their grooming behavior, potentially biasing the results. They also acknowledged they could not control for prior exposure to ticks, which can result in an immune response that affects the survival of later feeders.

## Forest Fragmentation and Biodiversity

Ostfeld suggests that fragmentation of forest habitat plays an important role in facilitating the spread of Lyme disease. His argument is based on the notion of nested biodiversity: Large swaths of habitat house diverse animal communities, and as forests are cleared for human use, species disappear from the remaining isolated scraps of habitat in a predictable sequence. This pattern has been documented on oceanic islands and other isolated habitats.^33^ But whether it applies to the forests of the northeastern and midwestern United States, where Lyme disease is most prevalent, remains a contentious issue.

**Figure:**
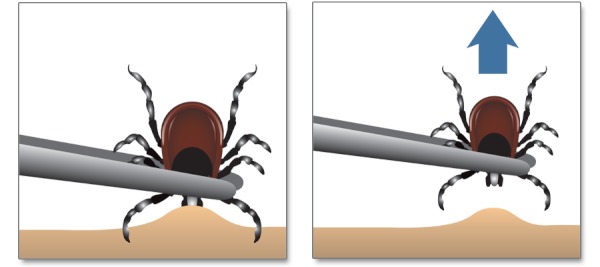
Tick Removal If you find a tick attached to your skin, there’s no need to panic. There are several tick-removal devices on the market, but a plain set of fine-tipped tweezers will remove a tick quite effectively. **How to remove a tick** Use fine-tipped tweezers to grasp the tick as close to the skin’s surface as possible.Pull upward with steady, even pressure. Don’t twist or jerk the tick; this can cause the mouth-parts to break off and remain in the skin. If this happens, remove the mouth-parts with tweezers. If you are unable to remove the mouth easily with clean tweezers, leave it alone and let the skin heal.After removing the tick, thoroughly clean the bite area and your hands with rubbing alcohol, an iodine scrub, or soap and water.Avoid folklore remedies such as “painting” the tick with nail polish or petroleum jelly or using heat to make the tick detach from the skin. Your goal is to remove the tick as quickly as possible—not wait for it to detach. **Followup** If you develop a rash or fever within several weeks of removing a tick, see your doctor. Be sure to tell the doctor about your recent tick bite, when the bite occurred, and where you most likely acquired the tick. Reprinted from: CDC. Tick Removal [website]. Atlanta, GA:Centers for Disease Control and Prevention (updated 15 Nov 2011). Available: http://www.cdc.gov/lyme/removal/index.html [accessed 12 Mar 2013].

Ostfeld’s work shows that the white-footed mouse, a powerful amplifier of Lyme disease risk, persists in small fragments of forest after other species disappear.^34^ He argues that hosts resistant to tick infestation and Bb infection are far more sensitive to human disturbance. Yet raccoons and opossums, which appear to be among the most effective dilution hosts for Bb, are common in urban and suburban areas. Studies from Illinois and California showed these animals thrived in remnants of forest and moved easily across farm fields.^33^^,^^35^ The California study noted that opossums prefer intensely disturbed habitats.

“If you fragment the forest, you still have all the main hosts for Bb,” says Maria Diuk-Wasser, a disease ecologist at Yale School of Public Health. “The major hosts are all human-adapted. Raccoons and opossums are present in people’s backyards.”

Diuk-Wasser is now collaborating with Fish on a study that tests the hypothetical link between biodiversity and human risk of Bb infection in new ways. Among the human residents of Block Island, off the coast of Rhode Island, Lyme disease is a common affliction. The island has low mammalian biodiversity; the only tick hosts present there are deer, white-footed mice, and birds. The researchers are trapping mice, collecting the ticks that infest them, and testing them for Bb infection. They’re cooperating with colleagues who have been collecting data on human cases of Lyme disease for years. The results from Block Island will be compared with those from a site on the Connecticut mainland, where a full complement of vertebrate tick hosts is present—and Lyme disease is also endemic.

If the dilution hypothesis holds, the number of infected nymphal ticks should be much higher on Block Island than on the mainland. The Yale investigators are also collecting ticks from backyards to directly examine the interface between humans and vectors of Bb. Fish, a critic of Ostfeld’s model of Bb ecology, does not expect to find simple correlations. “Community composition does affect Lyme disease ecology, but it’s not a rule of thumb that more biodiversity means less risk to people,” he says.

Both Ostfeld and Fish have coauthored studies that found a correlation between the size of forest habitats and the risk of Lyme disease. In surveys of 14 forest fragments ranging in size from 0.7 to 7.6 hectares, Ostfeld’s team found that white-footed mice were abundant in small forest patches and that the density of infected nymphal ticks was highest in the smallest patches (less than 1.2 hectares, comparable to the area inside an athletic track).^34^ Fish and his colleagues found a similar pattern in woodland habitats near Lyme, Connecticut, but noted that despite the higher number of infected ticks in fragmented habitats, the rate of human infections was lower there.^36^ This was so, the group concluded, because as woods were cleared for suburban development, the remaining habitat patches became few and far between, so that most people in the area never got near enough to a forest fragment to contact an infected tick.

## Connecting the Dots

It makes intuitive sense that more Bb-resistant hosts in the wild should lower the risk of human infection. But nothing about Lyme disease is simple. It turns out that even a species completely immune to Lyme infection can amplify the risk for people.

**Figure:**
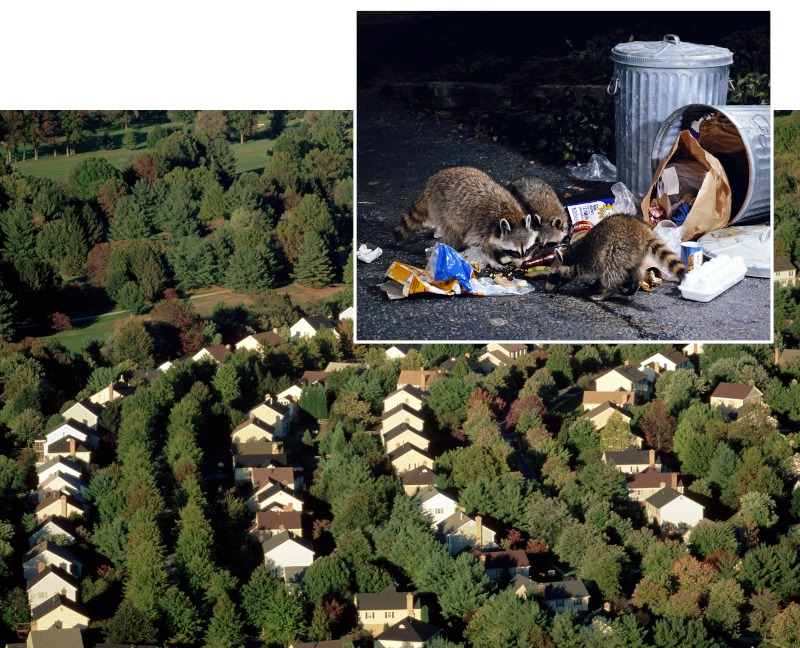
The relationship between forest fragmentation, biodiversity, and human risk of Lyme disease is still under debate—at least two research groups have shown that the density of Bb-infected ticks increased as habitat size shrank, yet one study showed that human infection rates went down at the same time. The role of urban-adapted “dilution hosts” such as raccoons and opossums remains unclear. Houses: © Steve & Dave Maslowski/Science Source; raccoons: © Paul A. Souders/Corbis

It boils down to a numbers game. The tick population depends on the presence of hosts to provide blood meals. If a Bb-resistant host species feeds enough larval ticks to lower the density of infected nymphs at the next life stage, it’s also likely to boost the overall tick population. That means more larvae will be around to feed on hosts that do pass along the disease, explains Randolph; the proportion of infected ticks may decline even as their abundance increases.

That phenomenon was illustrated in a recent experiment on Lyme disease ecology in California.^37^ The western fence lizard is an important host for disease-bearing ticks there but is resistant to Bb. Its immune response to the bacterium is so powerful that a lizard can actually clear the infection from the midgut of ticks feeding on it.^38^ This ability would seem to make the fence lizard the ultimate dilution host, and when researchers removed the lizards from test plots in oak woodlands, they expected the numbers of infected nymphal ticks to increase as a result. But the opposite occurred. The density of infected nymphs—and thus the potential risk of human infection—decreased in plots where lizards had been removed. The study, coauthored by Ostfeld, concluded that “the California Lyme disease system behaves differently than that in New York.”

In a critique of what she calls the “biodiversity-buffers-disease paradigm,” Randolph challenged the methodology and statistical analyses in Ostfeld’s work.^39^ She agrees that the dilution effect exists in nature, but she contends it is a rarity; depending on the circumstances in a given ecosystem, a greater diversity of vertebrate hosts may instead amplify the risk to humans. Furthermore, she says, it is not a simple changing index of biodiversity but specific changes in community structure that are critical to the outcome. Randolph is concerned that the dilution effect, shown to exist in a few specific situations for Lyme and other zoonotic diseases, is being used as an all-purpose argument for biodiversity conservation, which should be valued for other reasons. “The dilution effect is increasingly invoked but not well understood,” she says.

Recent studies have attempted to track a dilution effect for an array of infectious diseases, including schistosomiasis, West Nile virus, malaria, and hantavirus pulmonary syndrome. Ostfeld, Keesing, and others have cited the dilution effect as an example of a win–win solution for conservation and public health.^40^^,^^41^^,^^42^ Yet these authors acknowledge that the dilution effect doesn’t always hold and that many zoonoses emerge from regions of high biodiversity.

**Figure:**
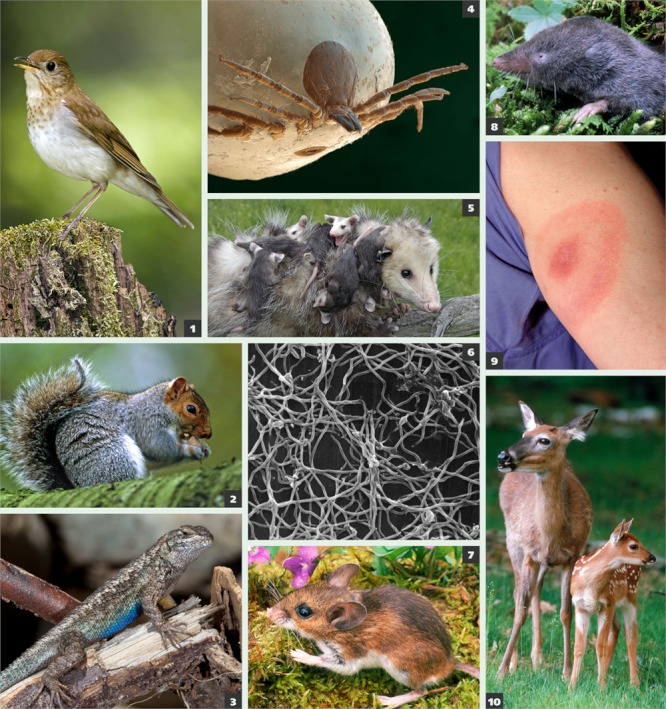
Some species, such as the white-footed mouse, are particularly efficient at passing Bb to the ticks that bite them. Others, such as the western fence lizard, actually clear Bb infection from biting ticks. Ultimately, the size of the tick population depends on the presence of hosts to provide blood meals. Bb-resistant host species may feed enough larval ticks to lower the proportion of infected nymphs at the next life stage. However, the overall numbers of ticks may be higher. In other words, the proportion of infected ticks may decline even as their abundance increases. Veery *(Catharus fuscescens)*© Jim Zipp/Science SourceGrey squirrel *(Sciurus carolinensis)*© Leslie J. Borg/Science SourceWestern fence lizard *(Sceloporus occidentalis)*© Stuart Wilson/Science SourceDeer tick *(Ixodes scapularis)*© Eye of Science/Science SourceOpossum *(Didelphis virginiana)*© Stephen J. Krasemann/Science SourceBorrelia burgdorferi© Stem Jems/Science SourceWhite-footed mouse *(Peromyscus leucopus)*© Gary Meszaros/Science SourceShort-tailed shrew *(Blarina brevicauda)*© Gary Meszaros/Science SourceHuman *(Homo sapiens)* with the distinctive bull’s-eye rash of Lyme disease© Science SourceWhite-tailed deer *(Odocoileus virginianus)*© Stephen J. Krasemann/Science Source

There are some striking examples of a link between biodiversity loss and increased human disease risk. *Schistosoma mansoni* is a parasite of freshwater snails that reproduces inside its snail host. It also infects humans through skin contact with a free-swimming larval stage. When overfishing depleted the population of snail-eating cichlid fishes in Lake Malawi, the incidence of schistosomiasis there rose.^41^^,^^43^ Experimental studies showed that adding snail species that resist the parasite lowered the infection rate in susceptible snails as well as the production of parasite larvae.^44^

However, this finding has not been tested in the field, and Chelsea Wood, a doctoral candidate in parasite and pathogen ecology at Stanford University, points out that schistosomiasis-infected snails don’t reproduce; their trematode parasites castrate them. “Reducing the infection rate among snails temporarily reduces schistosomiasis risk but soon leads to increases in the abundance of snails because fewer snails are castrated,” she explains. “The increased density of snails might lead to an increase in schistosomiasis risk, because even a small proportion of infections can mean a big risk if there are many snails.” Wood is lead author on a recent review that synthesizes different perspectives on the ecology of Lyme disease.^7^

In the case of West Nile virus, birds are the primary hosts. The infection is spread by mosquito vectors, including *Culex pipiens*, a species well adapted to urban environments.^45^ Some studies that examined human infection rates over broad geographic areas have found a correlation between increased diversity in bird communities and lowered rates of human infection.^46^^,^^47^ But a study of bird diversity in the Chicago area found no evidence of a dilution effect,^48^ and research in Connecticut found that *C. pipiens* mosquitoes preferred to take their blood meal from American robins.^45^ Regardless of whether robins are the most competent or abundant hosts, “they’re the most important because mosquitoes select them,” notes Diuk-Wasser, who coauthored the Connecticut study.

## Reaching Understanding

It has been written that without understanding the fundamental processes underlying the role of biodiversity in ecosystem functions and services, “attempts to forecast the societal consequences of biodiversity loss, and to meet policy objectives, are likely to fail.”^49^ Issues of both habitat type and landscape scale are important in understanding the ecology of zoonotic disease, be it Lyme disease or any other disease that has been cited as an example of the dilution effect. Bb is a forest-associated species, and the risk of human infection increases with proximity to forested landscapes. Within forests, however, biodiversity may buffer risk. Hantavirus shows a parallel pattern: It’s most prevalent in rodent communities of low diversity, but it’s a pathogen of rural areas. Despite low biodiversity in cities, you won’t catch hantavirus there.^7^

“Lyme disease is a poster child for the dilution effect,” says Wood. “Ecologists have extrapolated from research on Lyme to argue that biodiversity conservation can provide disease control for many other zoonotic diseases.- But if ecologists are going to suggest using biodiversity conservation to protect human health, we should at a minimum be sure that it won’t make the problem worse. The intense controversy shows we’re not sure of that—even for Lyme.”
